# Combined Use of Gefitinib and Bevacizumab in Advanced Non-Small-Cell Lung Cancer with *EGFR* G719S/S768I Mutations and Acquired C797S Without T790M After Osimertinib: A Case Report and Literature Review

**DOI:** 10.3390/curroncol32040201

**Published:** 2025-03-28

**Authors:** Wenting Lu, Jiayi Sun, Yawan Jing, Jing Xu, Chengming Huang, Yi Deng, Panwen Tian, Yalun Li

**Affiliations:** 1 Department of Respiratory and Critical Care Medicine, Integrated Care Management Center, Institute of Respiratory Health and Multimorbidity, West China Hospital, Sichuan University, Chengdu 610041, China; wentingluscu@163.com (W.L.); 13982172872@163.com (J.X.); hcm1911@163.com (C.H.); dygo6819@outlook.com (Y.D.); 2 Department of Pulmonary and Critical Care Medicine, State Key Laboratory of Respiratory Health and Multimorbidity, Institute of Respiratory Health and Multimorbidity, Institute of Respiratory Health, Frontiers Science Center for Disease-Related Molecular Network, Precision Medicine Center/Precision Medicine Key Laboratory of Sichuan Province, West China Hospital, Sichuan University, Chengdu 610041, China; sunjy0524@163.com (J.S.); 18076991158@163.com (Y.J.); mrascend@163.com (P.T.); 3 Lung Cancer Center/Lung Cancer Institute, West China Hospital, Sichuan University, Chengdu 610041, China

**Keywords:** NSCLC, *EGFR* mutation, *EGFR*-TKI, osimertinib, gefitinib, bevacizumab

## Abstract

Epidermal growth factor receptor (*EGFR*) tyrosine kinase inhibitors (TKIs) are effective in non-small-cell lung cancer (NSCLC) with sensitizing mutations. However, patients with uncommon *EGFR* mutations show variable responses, and resistance often develops. The C797S mutation is a common resistance mechanism after third-generation *EGFR*-TKI osimertinib therapy, with no standard treatment established. A 37-year-old Chinese woman with advanced NSCLC harboring *EGFR* G719S/S768I mutations developed an acquired C797S mutation without T790M after second- and third-generation *EGFR*-TKI therapy. She was treated with a combination of gefitinib and bevacizumab, achieving a partial response, particularly in liver metastases. Her overall survival exceeded 60 months. Gefitinib combined with bevacizumab demonstrates efficacy in managing NSCLC with uncommon *EGFR* mutations and overcoming acquired C797S resistance. This combination therapy offers a promising treatment strategy for patients with limited options after resistance to second- and third-generation *EGFR*-TKIs.

## 1. Introduction

Epidermal growth factor receptor (*EGFR*) mutations, a common oncogenic driver in non-small-cell lung cancer (NSCLC), can be targeted by *EGFR* tyrosine kinase inhibitors (TKIs) to improve patient prognosis. The *EGFR* exon 19 deletion and exon 21 L858R mutations are the most common activating and sensitizing *EGFR* mutations, accounting for 85–90% of *EGFR* mutation-positive cases [1]. Additionally, 10–15% of *EGFR* mutations are uncommon mutations [2], including *EGFR* 20ins, G719X, S768I, and L861Q. While limited information is available on the efficacy of *EGFR*-TKIs for these mutations, current clinical data suggest that they are more sensitive to second- and third-generation *EGFR*-TKIs than to first-generation *EGFR*-TKIs, excluding *EGFR* 20ins [3].

However, targeted therapy inevitably leads to acquired resistance. The most significant mechanism of resistance to first- and second-generation *EGFR*-TKIs is the acquired *EGFR* exon 20 p.T790M (T790M) [4]. Osimertinib, a third-generation *EGFR*-TKI, effectively targets the T790M mutation. However, following second-line treatment with osimertinib, 38% of patients developed resistance due to *EGFR* abnormalities, with the most common being the *EGFR* C797S mutation [5]. The T790M and C797S mutations in the *EGFR* gene are classified as either cis or trans based on their allelic relationship. The T790M-trans-C797S mutation responds to combined first- and third-generation *EGFR*-TKIs, whereas the T790M-cis-C797S mutation is resistant to all generations of *EGFR*-TKIs [6]. Based on previous research, patients with the C797S mutation may retain some sensitivity to first- or second-generation *EGFR*-TKI, such as gefitinib and afatinib [7]. The strategy to effectively overcome C797S-based osimertinib resistance involves fourth-generation *EGFR*-TKIs, most of which are currently in Phase I/II clinical trials. Therefore, there is no standard treatment for NSCLC patients with C797S mutation, particularly for those who develop this mutation without the T790M mutation after first-line therapy.

To date, there have been no reported cases of NSCLC with *EGFR* G719S/S768I/C797S triple mutations or their treatments. Here, we describe a patient diagnosed with stage IVB lung adenocarcinoma exhibiting uncommon *EGFR* mutations, G719S/S768I. After the progression of afatinib treatment, osimertinib was administered. Despite the absence of the T790M mutation, the C797S resistance mutation was identified. Ultimately, the combination of gefitinib and bevacizumab resulted in a partial response (PR) in the patient.

## 2. Case Presentation

In November 2019, a 37-year-old woman with no history of smoking was diagnosed with left lung adenocarcinoma with hilar mediastinal lymph node, left supraspinal lymph node, and extensive bone metastases (cT4N3M1c, stage IVB). Targetable mutation detection in tumor tissue using next-generation sequencing (NGS) by the GeneseeqPrime™ panel (Nanjing Geneseeq Technology Inc., Nanjing, China) [8] revealed *EGFR* exon 18 p.G719S mutation and *EGFR* exon 20 p.S768I mutation. The patient was administered with first-line afatinib (40 mg daily [qd] by mouth [po]) and subsequently developed new brain lesions (October 2020, Figure 1) after a 10-month period of stability. And the patient was then treated with second-line osimertinib (80 mg daily [qd] by mouth [po]). However, the patient’s brain lesions continued to deteriorate (February 2021, Figure 1). Due to the effectiveness of the third-generation *EGFR*-TKI treatment, third-line afatinib was reverted. Both brain and lung lesions remained stable for a duration of eight months. After developing resistance to afatinib, the patient exhibited disease progression in the brain lesions (November 2021, Figure 1), leading to the fourth-line almonertinib (220 mg daily [qd] by mouth [po]). After three months, the patient’s brain lesions showed increased progression (February 2022, Figure 1), prompting the initiation of fifth-line afatinib combined with pemetrexed. The patient expressed concerns regarding potential resistance to afatinib and requested a transition to osimertinib. After thorough evaluation and discussion, the clinical team concurred with proceeding with osimertinib as the subsequent line of therapy. The combination therapy resulted in stable disease (SD) and progression-free survival (PFS) of up to 21 months.

**Figure 1:**
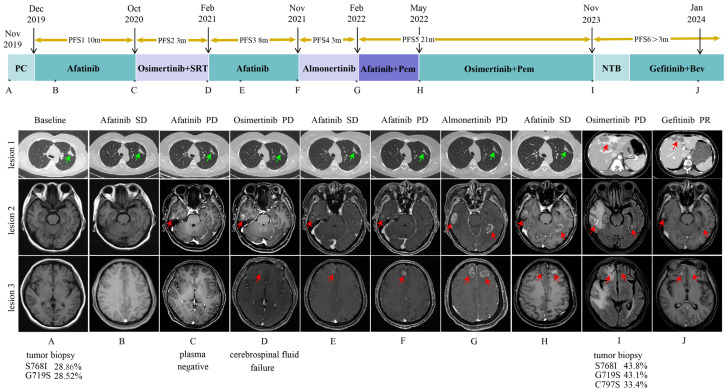
A summary of the patient’s clinical course. The upper panel shows the various treatments the patient received for *EGFR*-mutated non-small-cell lung cancer as well as the duration of each treatment. Asterisks indicate the time points for the response assessments. Green arrows indicate the primary lesion, while red arrows indicate the metastatic lesions. Genetic testing results for *EGFR* of the various tissue biopsies are shown below the corresponding CT images with their minor allele frequency. (**A**) Baseline chest CT scan and head MRI at diagnosis in November 2019. (**B**) SD on afitinib treatment in February 2020. (**C**) Lung lesion SD but head lesions PR on afitinib treatment in October 2020. (**D**) Lung lesion SD but head lesions PR on osimertinib treatment in February 2021. (**E**) SD on afitinib treatment in April 2021. (**F**) Lung lesion SD but head lesions PR on afitinib treatment in November 2021. (**G**) Lung lesion SD but head lesions PR on almonertinib in February 2022. (**H**) SD on afitinib treatment in May 2022. (**I**) Liver lesion and head lesion PD on osimertinib combined with pemetrexed treatment in November 2023. (**J**) PR on gefitinib and bevacizumab treatment in January 2024. Abbreviations: PFS, progression-free survival; PC, pemetrexed + cisplatin; SRT, stereotactic radiotherapy; Pem, pemetrexed; NTB, nab-paclitaxel + tirellizumab + bevacizumab; Bev, bevacizumab; SD, stable disease; PD, progressive disease; PR, partial response.

In November 2023, the patient developed multiple metastases in the chest wall, lungs, liver, and brain (Figure 1). Subsequently, a biopsy of a mass in the left chest wall confirmed the involvement of lung adenocarcinoma based on the pathological findings. NGS was performed with the patient’s tissue and revealed the retention of *EGFR* exon 18 p.G719S, *EGFR* exon 20 p.S768I, and the emergence of *EGFR* exon 20 p.C797S. Considering that patients may have developed resistance to osimertinib, the treatment was changed to gefitinib (250 mg daily [qd] by mouth [po]) combined with bevacizumab. After two months of treatment, the patient exhibited a significant reduction in the size of lung, brain, and liver metastases compared with their previous dimensions (January 2024, Figure 1). The lesions continued to show PR in June 2024. During combination therapy, the patient exhibited only grade 1 transaminase elevations, which improved with hepatoprotective treatment. No other adverse reactions, such as rash, proteinuria, or bleeding, were observed during this period. The patient continues to receive the combination therapy of gefitinib and bevacizumab. To date, the patient has achieved an overall survival (OS) of more than 60 months, with sustained clinical benefit and stable disease. The whole clinical course of treatment is shown in Figure 1. Antitumor responses were evaluated according to the Response Evaluation Criteria in Solid Tumors, version 1.1 (RECIST v1.1) [9].

## 3. Discussion

The *EGFR* T790M mutation alters the affinity of *EGFR* for ATP, significantly reducing the effectiveness of first- and second-generation *EGFR*-TKIs in competing for binding [10]. Osimertinib effectively targets both primary activating mutations and T790M resistance mutations [11]. However, resistance inevitably develops during treatment [12]. Common mechanisms of osimertinib resistance include the *EGFR* C797S mutation, *MET* amplification, T790M deletion, *BRAF* mutations, and *KRAS* mutations [13,14,15]. The *EGFR* C797S mutation is the primary cause of acquired resistance to osimertinib, often arising after the T790M mutation. Recently, C797S mutations have been documented in a few cases of T790M-negative patients following osimertinib treatment. It is well known that first-generation *EGFR*-TKIs are ineffective in patients with the T790M mutation. However, their efficacy in patients with C797S-positive and T790M-negative mutations remains uncertain. We summarized previous studies on the clinical outcomes of subsequent treatments in patients who developed C797S resistance mutations following osimertinib therapy [16,17,18,19,20]. In these cases, re-biopsies were performed on lung tissue, pleural effusion, plasma, and cerebrospinal fluid. The characteristics and clinical data of the patients are presented in Table 1. All patients received osimertinib and exhibited *EGFR* C797S-positive and T790M-negative resistance mutations. Notably, two patients with lung adenocarcinoma harboring the *EGFR* 19del and T790M mutations acquired the C797S mutation after failing osimertinib treatment and subsequently achieved partial remission with gefitinib.

Initially, we selected first-line targeted therapy with afatinib based on a pooled analysis of first-line studies showing favorable responses to afatinib in patients with G719X, S768I, and L861Q mutations, with a PFS comparable to that of common *EGFR* mutations [21,22]. Additionally, Passaro A et al. [23] demonstrated the promising efficacy of afatinib in a cohort of patients with brain metastases or rare *EGFR* mutations. In our study, the patient experienced therapeutic failure, as indicated by time to failure, after 10 and 8 months of afatinib treatment, consistent with findings from previous studies [21].

The patient in this case did not exhibit T790M mutation after progressing on afatinib treatment. However, the patient developed a C797S mutation during osimertinib treatment. The C797S mutation affects the cysteine residue at position 797 of the *EGFR* protein, and osimertinib demonstrated a strong antitumor effect by covalently binding to *EGFR* 797 cysteine [7,24]. Unfortunately, the C797S mutation blocks this binding, leading to further acquired resistance [25]. The case reported by Rangachari et al. [7,26] showed that in the presence of the C797S mutation alone, without T790M, resistant NSCLC patients may still retain sensitivity to gefitinib. The in vitro results presented by Niederst et al. [6] showed that *EGFR* Del19/C797S-positive and T790M-negative cell lines were resistant to third-generation *EGFR*-TKIs but remained sensitive to gefitinib. This may be due to the fact that the first-generation *EGFR*-TKI is primarily a reversible, non-selective inhibitor with a quinoline–amine structural motif that is not dependent on the cysteine at 797 to inhibit *EGFR* [24]. Consequently, first-generation *EGFR*-TKIs, such as gefitinib, still exert an inhibitory effect on the C797S mutation.

Studies have demonstrated that following the development of resistance to *EGFR*-TKIs, the level of tumor vascular endothelial growth factor (VEGF) increases [27], reducing tumor cell dependence on the *EGFR* signal and increasing dependence on the VEGF pathway. Preclinical studies have shown that an overactive VEGF/VEGFR pathway and tumor angiogenesis play a crucial role in the development of resistance to *EGFR*-TKIs. Bevacizumab, a monoclonal antibody that inhibits VEGF, has been utilized in various cancers due to its ability to suppress tumor angiogenesis. Studies indicate that bevacizumab can lead to significant improvements in PFS when used as a single agent, particularly in patients who cannot tolerate other chemotherapy options [28].

A previous Phase II clinical study showed that bevacizumab combined with first-generation *EGFR*-TKI significantly prolonged PFS in patients with NSCLC carrying *EGFR* mutations [29]. The combination of bevacizumab with an *EGFR*-TKI may enhance antitumor efficacy by targeting distinct pathogenic pathways, including angiogenesis and *EGFR* activation [30]. Additionally, in cases of tertiary C797S mutation, reintroducing a first-generation *EGFR*-TKI can still target the original sensitive *EGFR* mutation, even in the absence of T790M [16]. Thus, the dual targeting of the VEGF and EGFR pathways may effectively prevent drug resistance [31]. This is evidenced by our clinical case, which demonstrated the effectiveness of combining gefitinib, a first-generation *EGFR*-TKI, with bevacizumab in treating the *EGFR* C797S mutation following progression on the third-generation *EGFR*-TKI osimertinib.

Although gefitinib is typically well tolerated, it is associated with several adverse effects, including rash, diarrhea, and liver function abnormalities [32]. In addition, bevacizumab may lead to potential complications such as hypertension, bleeding, and gastrointestinal perforation [33]. The concomitant use of these two agents could increase the incidence of adverse events. Nevertheless, data from a Phase II clinical trial conducted in Japan indicated that serious adverse events associated with the combination of gefitinib and bevacizumab included grade 3 rash, hypertension, elevated aspartate aminotransferase and alanine aminotransferase, proteinuria, intracranial bleeding, and grade 4 gastrointestinal perforation [34]. Notably, no treatment-related deaths were reported. Fortunately, the patient in this case did not experience any of the serious adverse reactions, but close monitoring is still necessary during the combination therapy.

The *EGFR* C797S mutation is a key driver of resistance to third-generation *EGFR*-TKIs, making the development of next-generation inhibitors crucial for NSCLC treatment. Several fourth-generation *EGFR*-TKIs are currently in clinical trials. Although BLU-945 was one of the most advanced fourth-generation *EGFR*-TKIs [35,36], it was discontinued due to its dose-limiting toxicity and limited clinical benefit. EAI045, which targets the *EGFR* activator subunit, has proven ineffective as a monotherapy in blocking *EGFR*-driven cell proliferation, though it demonstrates significant antitumor activity in vitro and in animal models when combined with cetuximab [37,38]. Additionally, BDTX-1535, an orally bioavailable, potent, selective, and irreversible allosteric *EGFR* inhibitor, has shown promising preliminary efficacy and durability in Phase II trials in patients with relapsed or refractory *EGFR*-mutant NSCLC [39,40]. These agents offer novel therapeutic options with the potential to overcome resistance to third-generation *EGFR*-TKIs in NSCLC and other lung cancers.

For patients who develop a C797S mutation without a concurrent T790M mutation following treatment with *EGFR*-TKIs, the range of effective therapeutic options remains limited. In this case, while the patient exhibited PR to the combination of gefitinib and bevacizumab, the assessment of efficacy and the duration of treatment were relatively brief. It is therefore not possible to ascertain with certainty the precise efficacy of this combination therapy. Further studies with extended follow-up periods, along with overall survival and quality of life data, are required to evaluate the potential of this combination therapy in such cases.

## 4. Conclusions

In summary, we reported the clinical benefit of gefitinib combined with bevacizumab in a case characterized by *EGFR* G719S and S768I mutations/T790M negativity/C797S mutation-mediated resistance to osimertinib. Currently, treatment options for osimertinib-induced C797S mutation resistance are limited. This combination treatment may offer a potential new strategy.

## Figures and Tables

**Table 1:** Clinical characteristics and treatment outcomes of patients with NSCLC with acquired *EGFR* C797S after osimertinib resistance.

Article	Age/Gender	Smoking Status	Histologic Type	*EGFR* Mutation	*EGFR*-TKI	Response/PFS (m)	Re-Biopsy Specimen	Resistance Mutations	Second Treatment	Response/PFS (m)	Re-Biopsy Specimen	Resistance Mutations	Third Treatment	Response/PFS (m)
Chic N 2017 [18]	78/F	Never	AC	19Del	Afatinib	PR/30	Lesion biopsy	T790M	Osimertinib	PR/8	Lesion biopsy	T790M- C797S+	Gefitinib	PR/NA
Enrico D 2023 [14]	66/M	Former	AC	19Del, T790M	Osimertinib	PR/21	Body fluids	NA	Osimertinib	SD/8	Bronchoscopy	T790M- C797S+	Gefitinib	PR/4
Goldberg ME 2018 [15]	53/F	NA	AC	19Del	Afatinib	PR/8	Lesion biopsy	T790M	Osimertinib	PR/5	Lesion biopsy	T790M- C797S+	Osimertinib + Gefitinib	SD/5
Goldberg ME 2018 [15]	41/M	NA	AC	19Del	Afatinib	PR/23	Liquid biopsy	T790M	Osimertinib	PR/8	Lesion biopsy	T790M- C797S+	A cMet inhibitor (clinical trial)	NA
Russo A 2023 [16]	46/F	Never	AC	L858R	Osimertinib	PR/7	Lesion biopsy	C797S	Afatinib	PD/2	Liquid biopsy	C797S	Car + Gem	SD/3
Wang M 2021 [17]	48/F	NA	AC	19Del	Osimertinib	PR/18	Cerebrospinal fluid	C797S	Erlotinib + Pem + Cis +Bev	SD/4	NA	C797S	Erlotinib	SD/10

Abbreviations: NSCLC: non-small-cell lung cancer; *EGFR*, Epidermal growth factor receptor; *EGFR*-TKI, Epidermal growth factor receptor tyrosine kinase inhibitors; PFS, progression-free survival; PR, partial response; SD, stable disease; AC: adenocarcinoma; Pem: pemetrexed; Cis: cisplatin; Bev: bevacizumab; Car: carboplatin; Gem: gemcitabine; NA: not applicable.

## Data Availability

All data underlying the findings of this study are available within this publication. Patient data from the West China Hospital in Sichuan University have been anonymized to ensure confidentiality. Due to ethical and legal restrictions related to data protection regulations, raw patient data cannot be shared publicly. Requests for further information may be directed to the corresponding author.

## Abbreviations

The following abbreviations are used in this manuscript:

EGFR	Epidermal growth factor receptor
EGFR-TKIs	Epidermal growth factor receptor tyrosine kinase inhibitors
NSCLC	Non-small-cell lung cancer
PR	Partial response
NGS	Next-generation sequencing
SD	Stable disease
PFS	Progression-free survival
OS	Overall survival
VEGF	Vascular endothelial growth factor
